# Effects of COVID-19 Pandemic on Voluntary Medical Male Circumcision Services for HIV Prevention, Sub-Saharan Africa, 2020

**DOI:** 10.3201/eid2813.212455

**Published:** 2022-12

**Authors:** Megan E. Peck, Katherine S. Ong, Todd Lucas, Amber Prainito, Anne G. Thomas, Alex Brun, Valerian Kiggundu, Aisha Yansaneh, Lesego Busang, Kabelo Kgongwana, David Kelaphile, Khumo Seipone, Mpho H. Letebele, Panganai F. Makadzange, Amon Marwiro, Mirriam Sesinyi, Tyrone Lapidos, Njabuliso Lukhele, Vusi Maziya, Mandzisi Mkhontfo, Teruwork Gultie, Dejene Mulatu, Mesfin Shimelis, Tiruneh Zegeye, Tesfaye Teka, Marc Bulterys, John N. Njenga, Elijah Odoyo-June, Ambrose W. Juma, Leonard Soo, Norah Talam, Malerato Brown, Tafadzwa Chakare, Nyane Nonyana, Mpho A. Khoabane, Andrew F. Auld, Alice Maida, Wezi Msungama, Martin Kapito, Rose Nyirenda, Faustin Matchere, James Odek, Marcos Canda, Inácio Malimane, Jotamo Come, Nuno Gaspar, Antonio Langa, Mekondjo A. Aupokolo, Kaauma C. Vejorerako, Lawrence Kahindi, Denis Mali, Abeje Zegeye, Derek Mangoya, Brigitte L. Zemburuka, Jackson Bamwesigye, Ida Kankindi, Eugenie Kayirangwa, Samuel S. Malamba, Thierry Roels, Lenny Kayonde, Eugene Zimulinda, Emah Ndengo, Sabin Nsanzimana, Eric Remera, Gallican N. Rwibasira, Beata Sangwayire, Muhammed Semakula, Eugene Rugira, Eugene Rugwizangoga, Emmanuel Tubane, Emmanuel Yoboka, Joseph Lawrence, Dayanund Loykissoonlal, Nandi Maphothi, Victoria Achut, Sudhir Bunga, Monday Moi, Mbaraka Amuri, Kokuhumbya Kazaura, Daimon Simbeye, Neway Fida, Alick A. Kayange, Mohamed Seleman, Juliet Akao, Stella T. Alamo, Geoffrey Kabuye, Sheila Kyobutungi, Fredrick E. Makumbi, Peter Mudiope, Barbara Nantez, Omega Chituwo, Lingenda Godfrey, Brian Muyunda, Royd Kamboyi, Joseph Masiye, Eda Lifuka, John Mandisarisa, Mutsa Mhangara, Sinokuthemba Xaba, Carlos Toledo

**Affiliations:** US Centers for Disease Control and Prevention (CDC), Atlanta, Georgia, USA (M.E. Peck, K.S. Ong, T. Lucas. C. Toledo);; US Department of State, Washington, DC, USA (A. Prainito);; US Department of Defense, San Diego, California, USA (A.G. Thomas);; US Department of Defense, Washington, DC (A. Brun);; US Agency for International Development (USAID), Washington, DC (V. Kiggundu, A. Yansaneh);; African Comprehensive HIV/AIDS Partnerships, Gaborone, Botswana (L. Busang, K. Kgongwana, K. Seipone);; US Department of Defense, Gaborone (D. Kelaphile);; US CDC, Gaborone (M.H. Letebele, P.F. Makadzange);; Jhpiego, Gaborone (A. Marwiro);; Ministry of Health and Wellness, Gaborone (M. Sesinyi);; US Department of Defense, Mbabane, Eswatini (T. Lapidos);; USAID, Mbabane (N. Lukhele);; Ministry of Health, Mbabane (V. Maziya);; US CDC, Mbabane (M. Mkhontfo);; ICAP in Ethiopia, Addis Ababa, Ethiopia (T. Gultie);; Ministry of Health, Addis Ababa (D. Mulatu);; US CDC, Addis Ababa (M. Shimelis, T. Zegeye);; US Department of Defense, Addis Ababa (T. Teka);; US CDC, Nairobi, Kenya (M. Bulterys, J.N. Njenga, E. Odoyo-June);; Ministry of Health, Nairobi (Ambrose W. Juma);; USAID, Nairobi (L. Soo);; US Department of Defense, Nairobi (N. Talam);; US Department of Defense, Maseru, Lesotho (M. Brown);; Jhpiego, Maseru (T. Chakare, N. Nonyana);; Ministry of Health, Maseru (M.A. Khoabane);; US CDC, Lilongwe, Malawi (A.F. Auld, A. Maida, W. Msungama);; Ministry of Health, Lilongwe (M. Kapito, R. Nyirenda);; US Department of Defense, Lilongwe (F. Matchere);; USAID, Lilongwe (J. Odek);; US CDC, Maputo, Mozambique (M. Canda, I. Malimane);; Ministry of Health, Maputo (J. Come);; USAID, Maputo (N. Gaspar);; US Department of Defense, Maputo (A. Langa);; Ministry of Health and Social Services, Windhoek, Namibia (M.A. Aupokolo, K.C. Vejorerako);; Abt Associates Inc., Windhoek (L. Kahindi);; USAID, Windhoek (D. Mali, A. Zegeye);; Centre for HIV-AIDS Prevention Studies, Windhoek (D. Mangoya);; US CDC, Windhoek (B.L. Zemburuka);; US CDC, Kigali, Rwanda (J. Bamwesigye, I. Kankindi, E. Kayirangwa, S.S. Malamba, T. Roels);; US Department of Defense, Kigali (L. Kayonde, E. Zimulinda);; USAID, Kigali (E. Ndengo);; Rwanda Biomedical Center, Kigali (S. Nsanzimana, E. Remera, G.N. Rwibasira, B. Sangwayire, M. Semakula);; Alliance for Healthy Communities, Kigali (E. Rugira);; Jhpiego, Kigali (E. Rugwizangoga);; Ministry of Health, Kigali (E. Tubane, E. Yoboka);; USAID, Pretoria, South Africa (J. Lawrence);; Ministry of Health, Pretoria (D. Loykissoonlal);; US CDC, Pretoria (N. Maphothi);; Ministry of Health, Juba, South Sudan (V. Achut);; US CDC, Juba (S. Bunga);; US Department of Defense, Juba (M. Moi);; US CDC, Dar-es-Salaam, Tanzania (M. Amuri, K. Kazaura, D. Simbeye);; USAID, Dar-es-Salaam (N. Fida);; US Department of Defense, Dar-es-Salaam (A.A. Kayange, M. Seleman);; US Department of Defense, Kampala, Uganda (J. Akao);; US CDC, Kampala (S.T. Alamo, G. Kabuye);; USAID, Kampala (S. Kyobutungi);; Makerere University, Kampala (F.E. Makumbi, P. Mudiope);; Ministry of Health, Kampala (B. Nantez);; US CDC, Lusaka, Zambia (O. Chituwo, L. Godfrey, B. Muyunda);; Ministry of Health, Lusaka (R. Kamboyi, Jo. Masiye);; US Department of Defense, Lusaka (E. Lifuka);; US CDC, Harare, Zimbabwe (J. Mandisarisa); USAID, Harare (M. Mhangara);; Ministry of Health and Child Care, Harare (S. Xaba)

**Keywords:** COVID-19, 2019 novel coronavirus disease, coronavirus disease, severe acute respiratory syndrome coronavirus 2, SARS-CoV-2, viruses, respiratory infections, zoonoses, HIV prevention, impacts of COVID-19, voluntary medical male circumcision, HIV/AIDS, sub-Saharan Africa

## Abstract

Beginning in March 2020, to reduce COVID-19 transmission, the US President’s Emergency Plan for AIDS Relief supporting voluntary medical male circumcision (VMMC) services was delayed in 15 sub-Saharan African countries. We reviewed performance indicators to compare the number of VMMCs performed in 2020 with those performed in previous years. In all countries, the annual number of VMMCs performed decreased 32.5% (from 3,898,960 in 2019 to 2,631,951 in 2020). That reduction is largely attributed to national and local COVID-19 mitigation measures instituted by ministries of health. Overall, 66.7% of the VMMC global annual target was met in 2020, compared with 102.0% in 2019. Countries were not uniformly affected; South Africa achieved only 30.7% of its annual target in 2020, but Rwanda achieved 123.0%. Continued disruption to the VMMC program may lead to reduced circumcision coverage and potentially increased HIV-susceptible populations. Strategies for modifying VMMC services provide lessons for adapting healthcare systems during a global pandemic.

Voluntary medical male circumcision (VMMC, https://www.who.int/teams/global-hiv-hepatitis-and-stis-programmes/hiv/prevention/voluntary-medical-male-circumcision) has reduced HIV acquisition by ≈60% among men who engage in heterosexual sex and is an essential part of the Joint United Nations Program on HIV/AIDS strategy for ending AIDS by 2030 (*1*–*4*). In 2007, the World Health Organization and the Joint United Nations Program on HIV/AIDS recommended that countries in which prevalence of medical male circumcision was low and prevalence of HIV infection was high should be prioritized for VMMC (*5*). Countries originally prioritized were Botswana, Eswatini, Ethiopia, Kenya, Lesotho, Malawi, Mozambique, Namibia, Rwanda, South Africa, Tanzania, Uganda, Zambia, and Zimbabwe; South Sudan established a program in 2018 (*6*). Since the start of the program, the US President’s Emergency Plan for AIDS Relief (PEPFAR) has supported most VMMCs performed in prioritized countries (*7*). Most VMMCs are performed through conventional surgical methods, but some countries use device-based methods (*8*). VMMC programs provide a unique opportunity for men to access reproductive and sexual health services, beyond primary care, by providing a package of services that includes voluntary HIV testing, linkage to HIV care and treatment, and other HIV prevention services (*5*,*9*).

During 2008–2020, a total of 26.8 million VMMCs were performed in prioritized countries and were estimated to have averted 340,000 new HIV infections in men. Future population-level benefits are projected to be larger, including reductions of HIV infection up to 30%–40% in women (*10*,*11*). Timely VMMC among the priority age group, male clients 15–29 years of age, contribute to population-level HIV prevention benefits (*12*). However, depending on the length and severity of COVID-19, those population-level benefits could be affected (*13*).

Starting in March 2020, to minimize COVID-19 transmission risk, national governments instituted mitigation measures that led to the suspension or pausing of VMMC services. VMMC programs were vulnerable to the effects of mitigation measures, given that interventions such as suspending elective medical interventions and closing healthcare facilities directly affected services. Differences in demand for VMMC also resulted from changes in healthcare-seeking behavior; potential clients avoided healthcare settings because of the risk for nosocomial acquisition of disease. In addition, VMMC programs were affected by PEPFAR guidance released in April 2020, which recommended phasing out circumcisions among male clients 10–14 years of age (*14*). This change in guidance was prompted by an increase in reported adverse events among those 10–14 years of age and by modeling estimates demonstrating that the greatest reductions in HIV incidence were achieved by targeting men >15 years of age (*15*,*16*). The change in client age can potentially affect circumcision coverage given that, historically, the greatest proportion of VMMC male clients were 10–14 years of age.

Reports of the effects of the COVID-19 pandemic on public health services are limited, particularly in low-resource settings (*17*–*19*). To elucidate the effects of the COVID-19 pandemic on VMMC services, we compared VMMC services performed in 2020 with previous years among the 15 prioritized countries. Quantifying disruptions from the COVID-19 pandemic on a specific elective surgical procedure, VMMC, is valuable given that these procedures indicate pandemic-related disruptions to broader healthcare systems.

Collection of PEPFAR Monitoring, Evaluation and Reporting (MER) data is considered a public health program activity. The US Centers for Disease Control and Prevention Office of Human Research Protection Procedures determined that collection of MER data as nonresearch.

## Methods

To quantify the effects of the COVID-19 pandemic on VMMC services in US government fiscal year 2020, we analyzed key performance indicators from the MER database (*20*). All PEPFAR-supported VMMC programs report indicators quarterly in accordance with the US government fiscal year (October 1 through September 30 of the following year). Primary indicators reported include the total number of male clients circumcised and achievement of annual targets at the national level. Disaggregated indicators reported include VMMC performance by client age group and follow-up visit attendance (defined as client return for a postprocedure visit within 14 days of circumcision). To provide more information about the effects of the updated policy to phase out VMMC among clients <15 years of age, annual and quarterly VMMC results were reported separately for clients <15 and >15 years of age. We compared reported indicators in 2020 with those from 2016–2019 among all prioritized countries. We compared quarterly performance for the total number of nonmilitary VMMC sites and the number of clients per site across 14 countries for 2020. Nonmilitary VMMC sites are typically located at civilian health facilities, and military sites generally offer services at military facilities and target service members, their families, and the surrounding communities. We excluded from the site-level analyses military-supported VMMC sites because they do not report disaggregated VMMC indicators at the site level, as well as South Sudan because its program offers VMMC only at military sites.

To supplement the quantitative results, we conducted an exploratory review of narrative reports for April–June 2020. Programs submit narrative reports every quarter as one of the MER reporting requirements. Narrative reports provide an opportunity for programs to describe specific site-level issues that may have affected VMMC performance. We reviewed narrative reports to identify the following references to the effects of COVID-19 on VMMC services: national and local COVID-19 mitigation measures, efforts to maintain demand in VMMCs, and reallocation of resources. First, we reviewed narratives to broadly identify emergent themes across countries, and then we conducted a more thorough review in which countries were categorized into 1 of 3 impact levels. We analyzed narrative reports by using Microsoft Excel (https://www.microsoft.com) and used Stata 16 software (https://www.stata.com) for all other analyses. 

## Results

### VMMC Performance

The total number of VMMCs performed each year in the 15 prioritized countries decreased 32.5%, from 3,898,960 in 2019 to 2,631,951 in 2020 (Appendix Table 1). In most (11 of 15) countries, the total number of VMMCs performed was lower in 2020 than in 2019; mean percentage reduction was 49.4% (Appendix Table 2). The total number of annual VMMCs performed during 2016–2019 ranged by country from an average of >1,000 in South Sudan to >700,000 in Tanzania. Among 6 countries with larger programs (Kenya, Mozambique, South Africa, Tanzania, Uganda, and Zambia), performing on average of >250,000 VMMCs annually during 2016–2019, all countries except Zambia experienced a reduction of >100,000 VMMCs performed in 2020 compared with 2019. Among the larger programs, the largest reduction in performance was in South Africa; 513,631 VMMCs performed in 2019 decreased by 68.9% to 159,739 in 2020.

### Achievement of Annual Targets

Among all 15 countries combined, 66.7% of the 3,948,875 annual target (median 200,000, interquartile range 30,074–399,387) was met in 2020, compared with 102.0% of the 3,822,403 annual target in 2019 (median 145,035, interquartile range 31,884–430,986) (Appendix Table 1). The mean percentage achievement of annual targets was 62.2% in 2020 compared with 98.1% in 2019. In 2020, most (12 of 15) countries did not meet their annual national target (Appendix Table 2). Among the 12 countries that did not meet their national target in 2020, seven countries had either surpassed or achieved 90.0% of their annual target in 2019. Countries that exceeded their national annual targets in 2019 (South Africa by 101.0% and Zimbabwe by 104.9%) achieved less than half (30.7% and 42.6%, respectively) of their annual target in 2020.

### Quarterly Performance

The number of VMMCs performed during the first 2 quarters of fiscal year 2020 (October–December 2019 and January–March 2020) was 39.6% higher than the historic average during 2016–2019 (Appendix Table 1). During January–March 2020, just more than half (8 of 15) of countries increased the number of VMMCs performed compared with the same period in 2019; the average increase was 48.1% more VMMCs performed per country. However, during the early COVID-19 pandemic period, April–June 2020, the total number of VMMCs performed was 74.2% lower than it had been during the same period a year earlier. The number of VMMCs performed during April–June 2020 decreased in 13 countries, by 18.3% to 100.0%, compared with the same period in 2019. In South Africa, no PEPFAR-supported VMMCs were performed during April–June 2020.

**Figure 1:**
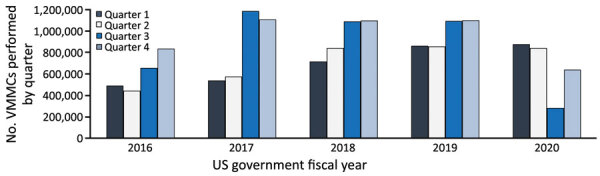
US President’s Emergency Plan for AIDS Relief–supported VMMCs performed in 15 countries in sub-Saharan Africa, by quarter and fiscal year (October 1–September 20), 2016–2020. VMMC, voluntary medical male circumcision.

Historically, most VMMCs, an average of 57.8% of the annual VMMC total targets in 2016–2019, are performed during April–September (Figure 1; Appendix Table 1). However, during April–September 2020, only 35.0% of the annual total number of VMMCs were performed. Although the number of VMMCs performed increased for most countries (12 of 15) during July–September 2020 compared with the previous period, most countries (8 of 15) performed fewer than half the number of VMMCs than they had in the corresponding period in 2019.

### Performance by Age Group

In 2020, a total of 26.7% of all VMMCs were performed for clients <15 years of age, representing a 56.2% decrease from the 41.1% performed for persons in this age group in 2019 (Appendix Table 1). After the PEPFAR policy to phase out VMMCs for clients <15 years of age was released in April 2020, eight countries did not report VMMCs among male clients 10–14 years of age during April–September 2020. The lowest number of VMMCs for male clients <15 years of age was reported during July–September 2020; 6.3% of all VMMCs were performed for male clients in this age group, compared with 40.3% in the same period in the previous year.

The annual total number of VMMCs performed for male clients >15 years of age was 1,879,201 in 2020 compared with 2,292,868 in 2019, representing a 18.0% decrease in the number of VMMCs performed in clients >15 years of age. During July–September 2020, most (94.3%) VMMCs were performed for clients >15 years of age, and the total volume of VMMCs conducted for persons in this age group was similar at 601,039, compared with 653,818 for the same period in 2019. However, clients >15 years of age contributed to the decline in achievement of PEPFAR annual targets, which decreased from 60.0% in 2019 to 47.6% in 2020.

### Performance at the Site Level

**Figure 2:**
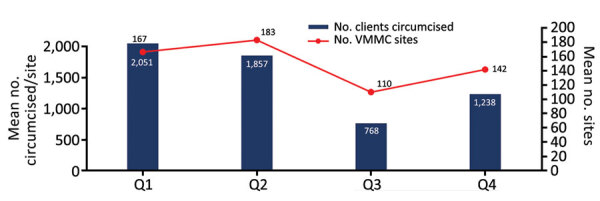
Mean number of male clients circumcised per US President’s Emergency Plan for AIDS Relief (PEPFAR)–supported nonmilitary VMMC site and mean number of PEPFAR VMMC sites, 14 countries in eastern and southern Africa prioritized for VMMC, by quarter, PEPFAR fiscal year 2020. Scales for the y-axes differ substantially to underscore patterns but do not permit direct comparisons. Q, quarter; VMMC, voluntary medical male circumcision.

Among the 14 countries reporting VMMCs at nonmilitary sites, the number of male clients circumcised at nonmilitary sites decreased 58.6%, from a mean of 1,857 clients/site during January–March 2020 to a mean of 768 clients/site during April–June (Figure 2). The number of nonmilitary sites performing VMMCs per country declined 39.3%, from a mean of 183 sites during January–March to a mean of 110 during April–June 2020. Although the total number of nonmilitary sites performing VMMCs increased 29.1% during 2020, from a mean of 110 during April–June to 142 during July–September, this increase remained 19.1% below the mean number of nonmilitary sites performing VMMCs during October 2019–March 2020.

### Follow-up Visits

Among all 15 countries, the overall proportion of VMMC clients who had a postprocedure follow-up visit increased from 89.6% in 2019 to 91.1% in 2020. Eight countries reported a decreased rate of follow-up visits in 2020 compared with the previous year, and 7 countries reported either the same rate of follow-up or an increase, ranging from 2.2% to 19.1%. The rate of client follow-up stayed the same, at 93% during October 2019–June 2020, and decreased to 88% during July–September 2020.

### VMMC Narrative Reports

Our review of the narratives indicated that service disruptions varied from minimal to major. Countries categorized as minimally affected reported only slight modifications to their program and minor reductions in performance. Moderately affected programs reported suspending services for at least 1–2 months, and majorly affected programs reported suspending services for >2 months. Zambia and South Sudan were identified as having minimally disrupted programs, and although there were service disruptions mentioned in the narrative reports, overall performance was only somewhat affected. In South Sudan, services continued with restrictions on the number of clients allowed in the waiting area. Moderately disrupted programs had service suspensions during April–June but eventually resumed offering VMMCs to clients who sought services through walk-in visits; however, outreach efforts to recruit clients stopped. Among all countries, the most commonly reported mitigation measures that reduced VMMC services were curfews, shelter-in-place orders, and physical distancing requirements.

VMMC narrative reports also described redirecting efforts to support and assist local service providers with COVID-19–related activities. Those efforts shifted the focus of programs, and many diverted staff to support COVID-19 response, particularly during the early part of the pandemic (*21*). Other VMMC staff were used to support essential HIV clinical services (e.g., treatment services). In addition, programs adapted demand creation strategies, which included logging clients interested in VMMC, booking clients for the next month, using COVID-19 contact tracers to also serve as VMMC mobilizers, and disseminating messages through community campaigns.

Countries that continued to offer VMMCs reported adjusting services to minimize risk for COVID-19 transmission. Adjustments included telescreening, space modifications to manage client flow, prior registration of clients, appointment-only visits, extended hours, and restricted numbers of clients allowed at a site per day. In some countries, to comply with restrictions in movements and curfews,VMMC sites were offered only at health facilities located in communities. Infection prevention measures included distributing personal protective equipment to staff, screening clients for COVID-19, and sanitizing spaces.

## Discussion

VMMC is part of countries’ HIV prevention portfolio and can contribute to HIV epidemic control (*22*,*23*). To maximize these public health benefits, country-specific VMMC program targets are established. However, because of the COVID-19 pandemic, most countries prioritized for VMMC did not reach these targets in 2020. A global target of 25 million VMMCs during 2016–2020 and 41.5 million by 2030 has been endorsed by countries prioritized for VMMC (*24*). Substantial progress has been made toward these targets, but those gains have been affected by the COVID-19 pandemic.

Overall, the VMMC program has grown substantially since 2016; mean annual increase in the number of VMMCs performed during 2016–2019 is 20.7%. VMMC performance before the COVID-19 pandemic (October 2019–March 2020) indicated successful scale-up of the program and that performance was on track to outperform the previous year. However, the number of VMMCs performed was substantially lower during April–September 2020 than during the previous year; decreases were especially pronounced during April–June 2020. These patterns are consistent with known COVID-19 mitigation measures, particularly in the early phase of the pandemic.

Reductions in the number of VMMCs performed during 2020 were similar to disruptions of other elective surgical procedures. In the United States, one study found that the overall rate of elective surgical procedures decreased 48.0% during the initial shutdown of elective procedures in March 2020 compared with the previous year (*25*). In Ethiopia, a study found that elective surgeries dropped by 32.0% at a major hospital after the COVID-19 pandemic was declared (*26*). Compared with disruptions to other HIV prevention services (e.g., preexposure prophylaxis), VMMC services were more disrupted and time to service resumption was longer (*27*).

PEPFAR recommends that VMMC programs conduct COVID-19 readiness assessments to determine if sites can reopen. By the end of September 2020, all countries had resumed VMMC services. However, the rate of resumption was not uniform, most likely because of varying COVID-19 epidemiology and differences in response policies (*28*). Countries such as South Africa experienced severe effects, given that they reported some of the highest COVID-19 case counts on the continent, prompting ongoing national COVID-19 mitigation measures (*29*–*31*). Zimbabwe was also affected by high COVID-19 cases counts and subsequent national lockdowns (*32*).

One success for VMMC programs in 2020 was implementation of the new PEPFAR guidance to stop providing VMMCs for clients <15 years of age. This success is demonstrated by performance of 11.3% of VMMCs for clients 10–14 years of age during April–June 2020, compared with 41.0% during the same period in 2019. Implementation of the updated PEPFAR guidance released in April 2020 varied across countries; clients <15 years of age continued to be circumcised throughout 2020. This variability probably resulted from some countries continuing to perform device-based circumcisions for clients 13–14 years of age, given that a World Health Organization prequalified medical device has been approved for use in this age group. Although overall the number of VMMCs conducted for clients >15 years of age decreased in 2020 compared with 2019, the number of VMMCs conducted for clients in this age group was the highest during July–September, compared with the same period during 2016–2018. This increase probably directly resulted from programs adapting demand creation strategies to target older clients. Follow-up visits may have potentially increased in 2020 compared with 2019 because programs started conducting virtual follow-up visits when in-person visits were not safe or feasible.

Narrative reports from April–June 2020 suggest that governments enacted various types of COVID-19 mitigation measures given the different public health needs, priorities, and resources across countries. Although these measures presented new challenges to reaching HIV epidemic control targets, they also provided opportunities for improvements. Innovative approaches that enabled continuation of VMMC services included programs offering virtual follow-up visits, using web-based mobile apps for real time reporting, increasing flexibility for clients who prefer off-hour services, and adopting practices to avoid overcrowding.

Lessons learned from the COVID-19 pandemic can be used to inform other elective surgical and prevention services and to update VMMC policies and guidelines to help prepare for future global and national emergencies. Mechanisms for rapid availability and allocation of funding to safely resume services and carry over unused funds can be established. We recommend greater financial support for programmatic components, such as demand creation activities that can be safely implemented during the pandemic, including adaptating social and digital media platforms. These activities may be more costly than traditional in-person mobilization efforts. Those costs are a result of extended site hours, expanded numbers of VMMC sites, and increased transportation to sites to limit the number of clients in a vehicle. Support to maintain VMMC services during the COVID-19 pandemic is particularly valuable, given that modeled estimates have demonstrated that, in certain settings, the risk for death from HIV may be far greater than the risk for death from COVID-19 (*33*).

The pandemic will probably have long-term effects on VMMC program practices; country reports describe challenges with VMMC-eligible men expressing concerns about visiting public health facilities for fear of contracting COVID-19 or overburdening healthcare services (*34*). Factors that could continue to keep VMMC programs from resuming services to prepandemic levels include ongoing travel restrictions, varying global COVID-19 epidemiology, and low COVID-19 vaccination coverage.

Of the 4 limitations to our study, the first is the limitation to PEPFAR program data, which captured the timing of pausing and resuming VMMC services on a quarterly basis only. Thus, temporal trends between mitigation measures or national COVID-19 epidemiology and decisions to pause services could not be described at a weekly or monthly level. Second, COVID-19 mitigation measures were not uniformly implemented across or within all countries, and the extent to which these measures were executed was not included in this analysis. Third, the findings from this analysis are limited to PEPFAR-supported sites in 15 countries in eastern and southern Africa. Last, this analysis was ecologic, and the effect of the COVID-19 pandemic on VMMC in 2020 cannot be distinguished from other effects such as changes in client age eligibility.

To optimize the HIV prevention benefits of VMMC, steps can be taken to increase program resilience to ensure that HIV prevention interventions are able to quickly adapt to public health emergencies, particularly to COVID-19 mitigation measures. These efforts are relevant given that the severity of the effect of COVID-19 in countries prioritized for VMMC remains unknown. If healthcare systems are not able to maintain HIV prevention programs while managing the response to the COVID-19 pandemic, they are likely to experience increasing HIV incidence.

Appendix Supplemental results for study of effects of COVID-19 pandemic on voluntary medical male circumcision services for HIV prevention, sub-Saharan Africa, 2020.
