# Supplementary material for: Effects of COVID-19 Pandemic on Voluntary Medical Male Circumcision Services for HIV Prevention, Sub-Saharan Africa, 2020
Source: Emerg Infect Dis. 2022 Dec;28(Suppl 1):S262–9. doi: 10.3201/eid2813.212455 (PMC9745243; doi:10.3201/eid2813.212455)
Supplement: Appendix — Supplemental results for study of effects of COVID-19 pandemic on voluntary medical male circumcision services for HIV prevention, sub-Saharan Africa, 2020. [file 21-2455-Techapp-s1.pdf]

Article DOI: https://doi.org/10.3201/eid2813.212455

Effects of COVID-19 Pandemic on Voluntary Medical Male Circumcision Services for HIV Prevention, Sub-Saharan Africa, 2020

Appendix

Table 1. Annual results of PEPFAR-funded voluntary medical male circumcision indicators, among 15 prioritized countries in sub-Saharan Africa, fiscal years, 2016–2020*

Fiscal year	VMMCs performed, no. (%)	Difference in no. VMMCs performed compared with the prior year, %	Annual PEPFAR program targets	Achievement of PEPFAR annual target, %	VMMCs performed, by quarter, no. (%)			
					Quarter 1 (Oct–Dec)	Quarter 2 (Jan–Mar)	Quarter 3 (Apr Jun)	Quarter 4 (Jul–Sep)
Total	15,928,896	NA	17,769,866	89.6†	3,459,689 (21.7)‡	3,539,253 (22.2)‡	4,274,494 (26.8)‡	4,656,043 (29.2)‡
2016	2,293,001 (14.4)	NA	2,452,414	93.5	485,067 (21.2)‡	438,130 (19.1)‡	640,017 (27.9)‡	730,585 (31.9)‡
2017	3,383,949 (21.2)	47.6	3,662,622	92.4	533,611 (15.8)‡	570,772 (16.9)‡	1,180,204 (34.9)‡	1,099,380 (32.5)‡
2018	3,721,035 (23.4)	9.9	3,883,552	95.8	707,191 (19.0)‡	838,115 (22.5)‡	1,083,353 (29.1)‡	1,092,343 (29.4)‡
2019	3,898,960 (24.5)	4.8	3,822,403	102.0	859,987 (22.1)‡	852,995 (21.9)‡	1,089,946 (28.0)‡	1,096,044 (28.1)‡
2020	2,631,951 (16.5)	−32.5	3,948,875	66.7	873,833 (33.2)‡	839241 (31.9)‡	280,974 (10.7)‡	637,691 (24.2)‡
Clients <15 y§	6,554,675 (41.1)	NA	NA	36.9	1,436,402 (41.5)¶	1,410,195 (39.8)¶	1,681,811 (39.3)¶	1,878,675 (40.3)¶
2016	1,030,182 (44.9)	NA	NA	42.0	201,577 (41.6)¶	147,551 (33.7)¶	260,532 (40.7)¶	418,406 (57.3)¶
2017	1,523,520 (45.0)	47.8	NA	41.6	194,132 (36.4)¶	268,188 (47.0)¶	458,986 (38.9)¶	504,432 (45.9)¶
2018	1,694,318 (45.5)	11.2	NA	43.6	327,115 (46.3)¶	371,303 (44.3)¶	483,932 (44.7)¶	474,470 (43.4)¶
2019	1,604,016 (41.1)	−5.3	NA	42.0	359,558 (421.8)¶	353,563 (41.4)¶	446,653 (41.0)¶	441,202 (40.3)¶
2020	702,639 (26.7)	−56.2	NA	17.8	354,020 (40.5)¶	269,590 (32.1)¶	31,708 (11.3)¶	40,165 (6.3)¶
Clients ≥15 y§	9,275,813 (58.2)	NA	NA	52.2	1,881,874 (54.4)¶	1,957,238 (55.3)¶	2,404,293 (56.2)¶	2,820,654 (60.6)¶
2016	1,108,891 (48.4)	NA	NA	45.2	232,912 (48.0)¶	190,692 (43.5)¶	298,833 (46.7)¶	455,115 (62.3)¶
2017	1,866,041 (55.1)	68.3	NA	50.9	280,204 (52.5)¶	324,033 (56.8)¶	632,494 (53.6)¶	515,548 (46.9)¶
2018	2,128,812 (57.2)	14.1	NA	54.8	357,005 (50.5)¶	438,577 (52.3)¶	577,144 (53.3)¶	595,134 (54.5)¶
2019	2,292,868 (58.8)	7.7	NA	60.0	492,102 (57.2)¶	498,579 (58.5)¶	643,157 (59.0)¶	653,818 (59.7)¶
2020	1,879,201 (71.4)	−18.0	NA	47.6	519,651 (59.5)¶	505,357 (60.2)¶	252,665 (89.9)¶	601,039 (94.3)¶

*Fiscal years, October 1–September 30. VMMC, voluntary medical male circumcision; NA, not applicable; PEPFAR, US President's Emergency Plan for AIDS Relief.
†Among the combined annual PEPFAR program targets from 2016 - 2020
‡Totals do not equal annual cumulative total because reported disaggregated totals by age are slightly less (90%–95%).
§Among annual no. of PEPFAR supported VMMCs performed
¶Among the quarterly sum of no. of PEPFAR supported VMMCs performed in corresponding year

Table 2. US President's Emergency Plan for AIDS Relief–supported VMMCs performed, by country, fiscal years 2019–2020, by age category and achievement of annual targets, among 15 sub-Saharan African countries*

							Quarter 1 (October - December)		Quarter 2 (Jan–Mar)		Quarter 3 (Apr–Jun)		Quarter 4 (Jul– Sep)	
Country	Fiscal year	Annual target	VMMCs performed, no. (% achievement of annual target)	Change in no. VMMCs performed in 2020 vs. 2019, %	Client age <15 y, %	Client age ≥15 y, %	No. (%)	Change in VMMCs performed in 2020 vs. 2019, %	No. (%)	Change in VMMCs performed in 2020 vs. 2019, %	No. (%)	Change in VMMCs performed in 2020 vs. 2019, %	No. (%)	Change in VMMCs performed in 2020 vs. 2019, %
Botswana	2019	26,301	21,071 (80.1)	−64.6	11,398 (54.1)	9,673 (45.9)	5,778 (27.4)	−17.4	2,886 (13.7)	−65.3	4,287 (20.3)	−95.1	8,120 (38.5)	−81.9
	2020	25,876	7,449 (28.8)		3,279 (44.0)	4,147 (55.7)	4,771 (64.0)		1,001 (13.4)		208 (2.8)		1,469 (19.7)	
Eswatini	2019	30,000	15,127 (50.4)	−39.2	8,839 (58.4)	6,284 (41.5)	1,712 (11.3)	37.4	2,277 (15.1)	108.8	5,128 (33.9)	−84.0	6,010 (39.7)	−78.9
	2020	29,989	9,197 (30.7)		4,113 (44.7)	5,084 (55.3)	2,353 (25.6)		4,755 (51.7)		819 (8.9)		1,270 (13.8)	
Ethiopia	2019	31884	26,631 (83.5)	30.6	11,560 (43.4)	15,071 (56.6)	2,440 (9.2)	200.3	10,184 (38.2)	1.4	6,752 (25.4)	9.2	7,255 (27.2)	34.5
	2020	45596	34,786 (76.3)		3,519 (10.1)	31,267 (89.9)	7,327 (21.1)		10,326 (29.7)		7,373 (21.2)		9,760 (28.1)	
Kenya	2019	300,051	281,038 (93.7)	−56.7	210,483 (74.9)	70,555 (25.1)	135,164 (48.1)	−54.6	25,917 (9.2)	−39.5	56,929 20.3)	−77.5	63,028 (22.4)	−49.6
	2020	200,000	121,576 (60.8)		63,630 (52.3)	57,907 (47.6)	61,338 (50.5)		15,669 (12.9)		12,806 (10.5)		31,763 (26.1)	
Lesotho	2019	36719	29,151 (79.4)	−69.6	17,079 (58.6)	12,071 (41.4)	5,742 (19.7)	−17.9	7,559 (25.9)	−50.1	8,353 (28.7)	−95.8	7,497 (25.7)	−99.7
	2020	30074	8,861 (29.5)		2,622 (29.6)	6,239 (70.4)	4,716 (53.2)		3,770 (42.5)		353 (4.0)		22 (0.2)	
Malawi	2019	145,035	139,128 (95.9)	−51.2	43,378 (31.2)	95,748 (68.8)	24,922 (17.9)	−13.3	14,786 (10.6)	95.7	34,150 (24.5)	−96.0	65,270 (46.9)	−85.6
	2020	206,396	67,943 (32.9)		11,979 (17.6)	55,964 (82.4)	28,249 (41.6)		28,932 (42.6)		1,381 (2.0)		9,381 (13.8)	
Mozambique	2019	430,986	390,498 (90.6)	−51.1	197,823 (50.7)	192,675 (49.3)	66,068 (16.9)	41.7	89,078 (22.8)	2.7	104,407 (26.7)	−98.0	130,945 (33.5)	−97.2
	2020	392,015	190,895 (48.7)		89,705 (47.0)	101,190 (53.0)	93,600 (49.0)		91,508 (47.9)		2,064 (1.1)		3,723 (2.0)	
Namibia	2019	54,306	40,465 (74.5)	−33.8	17,499 (43.2)	22,333 (55.2)	4,393 (10.9)	−31.6	4,322 (10.7)	−16.1	14,594 (36.1)	−19.7	17,156 (42.4)	−50.8
	2020	50,203	26,784 (53.4)		7,345 (27.4)	19,439 (72.6)	3,005 (11.2)		3,626 (13.5)		11,719 (43.8)		8,434 (31.5)	
Rwanda	2019	107,234	133,797 (124.8)	82.4	39,208 (29.3)	94,589 (70.7)	36,755 (27.5)	51.3	25,669 (19.2)	119.3	49,715 (37.2)	−18.3	21,658 (16.2)	322.2
	2020	198,440	243,998 (123.0)		44,059 (18.1)	199,939 (81.9)	55,627 (22.8)		56,292 (23.1)		40,641 (16.7)		91,438 (37.5)	
South Africa	2019	508,645	513,631 (101.0)	−68.9	222,541 (43.3)	289,875 (56.4)	127,772 (24.9)	23.4	104,768 (20.4)	−26.9	173,935 (33.9)	−100.0	107,156 (20.9)	−96.6
	2020	519,998	159,739 (30.7)		42,550 (26.6)	117,169 (73.4)	97,907 (61.3)		58,143 (36.4)		0 (0.0)		3,689 (2.3)	
South Sudan	2019	1,031	1,461 (141.7)	19.2	27 (1.8)	1,434 (98.2)	365 (25.0)	−2.2	453 (31.0)	9.7	305 (20.9)	52.8	338 (23.1)	24.6
	2020	1,545	1,741 (112.7)		35 (2.0)	1,706 (98.0)	357 (20.5)		497 (28.5)		466 (26.8)		421 (24.2)	
Tanzania	2019	748,738	778,084 (103.9)	−30.2	306,254 (39.4)	471,830 (60.6)	144,219 (18.5)	25.1	232,661 (29.9)	−13.7	169,349 (21.8)	−76.6	231,855 (29.8)	−47.2
	2020	749,356	543,194 (72.5)		233,293 (42.9)	309,883 (57.0)	180,437 (33.2)		200,849 (37.0)		39,567 (7.3)		122,341 (22.5)	
Uganda	2019	756,456	727,548 (96.2)	−17.2	260,484 (35.8)	466,950 (64.2)	149,178 (20.5)	13.8	189,490 (26.0)	−2.6	212,425 (29.2)	−73.3	176,455 (24.3)	8.6
	2020	800,000	602,414 (75.3)		147,273 (24.4)	455,102 (75.5)	169,706 (28.2)		184,471 (30.6)		56,624 (9.4)		191,613 (31.8)	
Zambia	2019	338,874	480,257 (141.7)	1.0	126,920 (26.4)	353,294 (74.0)	95,843 (20.0)	15.0	77,751 (16.2)	44.7	156,328 (32.6)	−32.7	150,335 (31.3)	4.6
	2020	399,387	485,243 (121.5)		49,760 (10.3)	435,483 (89.7)	110,235 (22.7)		112,518 (23.2)		105,256 (21.7)		157,234 (32.4)	
Zimbabwe	2019	306,143	321,085 (104.9)	−60.1	130,526 (40.7)	190,485 (59.3)	59,636 (18.6)	−9.1	65,194 (20.3)	2.6	93,289 (29.1)	−98.2	102,966 (32.1)	−95.0
	2020	300,000	127,919 (42.6)		49,237 (38.5)	78,682 (61.5)	54,205 (42.4)		66,884 (52.3)		1,697 (1.3)		5,133 (4.0)	

*VMMC, voluntary medical male circumcision.
